# Anthelmintic Effect of *Leucaena leucocephala* Extract and Its Active Compound, Mimosine, on Vital Behavioral Activities in *Caenorhabditis elegans*

**DOI:** 10.3390/molecules27061875

**Published:** 2022-03-14

**Authors:** Amal Widaad, Ihsan Nazurah Zulkipli, Mark I. R. Petalcorin

**Affiliations:** PAPRSB Institute of Health Sciences, Universiti Brunei Darussalam, Jalan Tungku Link, Gadong BE1410, Brunei or 17h0176@ubd.edu.bn (A.W.); nazurah.zulkipli@ubd.edu.bn (I.N.Z.)

**Keywords:** anthelmintic, *Leucaena leucocephala*, mimosine, *Caenorhabditis elegans*, head thrashing, egg-laying, pharyngeal pumping, EGL-19

## Abstract

Helminth infections continue to be a neglected global threat in tropical regions, and there have been growing cases of anthelmintic resistance reported towards the existing anthelmintic drugs. Thus, the search for a novel anthelmintic agent has been increasing, especially those derived from plants. *Leucaena leucocephala* (LL) is a leguminous plant that is known to have several pharmacological activities, including anthelmintic activity. It is widely known to contain a toxic compound called mimosine, which we believed could be a potential lead candidate that could exert a potent anthelmintic effect. Hence, this study aimed to validate the presence of mimosine in LL extract and to investigate the anthelmintic effect of LL extract and mimosine on head thrashing, egg-laying, and pharyngeal pumping activities using the animal model *Caenorhabditis elegans (C. elegans).* Mimosine content in LL extract was confirmed through an HPLC analysis of spiking LL extract with different mimosine concentrations, whereby an increasing trend in peak heights was observed at a retention time of 0.9 min. LL extract and mimosine caused a significant dose-dependent increase in the percentage of worm mortality, which produced LC50s of 73 mg/mL and 6.39 mg/mL, respectively. Exposure of *C. elegans* to different concentrations of LL extract and mimosine significantly decreased the head thrashing, egg-laying, and mean pump amplitude of pharyngeal pumping activity. We speculated that these behavioral changes are due to the inhibitory effect of LL extract and mimosine on an L-type calcium channel called EGL-19. Our findings provide evidential support for the potential of LL extract and its active compound, mimosine, as novel anthelmintic candidates. However, the underlying mechanism of the anthelmintic action has yet to be elucidated.

## 1. Introduction

Parasitic or helminth infections have been a major global health issue with significant morbidity infecting over 1.5 billion worldwide [1,2]. Ruminant animals infected with parasites develop growth retardation, low milk production, weight loss, impaired reproduction, and even mortality [3,4,5]. In humans, serious infections lead to anemia and intestinal blockage, while impaired cognitive and physical development are associated with infections in children [6,7,8]. In addition, helminth infections have a significant impact on the socio-economic state. The burden of these parasitic diseases is attributable to poverty, poor hygiene, and a lack of proper sanitation [6,9].

The significant problem imposed by helminth infections has led to increased efforts in parasitic control and preventive and treatment regimes. These strategies generally focus more on the use of synthetic anthelmintic drugs. However, the parasitic worms have continuously developed anthelmintic resistance towards these drugs [10,11,12,13,14]. Several integrated techniques have been conducted to overcome this issue, including pasture management, gene manipulation, change of feeding nutrition, and natural products with anthelmintic properties [15].

The use of naturally derived dewormers from herbal plants has long been considered, mainly because of their effectiveness, availability, and low toxicity [4,16,17]. Many medicinal plants exhibited anthelmintic activity, including *Leucaena leucocephala* (LL). LL is a leguminous plant under the *Fabaceae* family and subfamily *Mimosoideae* [18]. It is addressed differently in several countries, such as ‘White lead tree’ in America, ‘Ipil-ipil’ in the Philippines, and ‘Petai belalang’ in Malaysia [19]. It is also infamously known as the ‘miracle tree’ for its wide range of uses and benefits, including as ruminant feeds, for controlling soil erosion, and the production of timber and fuelwood [18,20]. Various studies have demonstrated several pharmacological activities exerted by this plant, including anti-bacterial [21,22,23,24], anti-inflammatory [25,26], anti-cancer [27,28,29], and anthelmintic activities [30,31,32,33], and it is believed to be due to the different types of phytochemicals present in the plant, such as alkaloids, flavonoids, tannins, and quercetin. However, the use of this plant as an animal feed could not be fully potentiated due to the presence of a toxic compound called mimosine that has been reported to cause alopecia, hair loss, growth retardation, and infertility in ruminants [34,35,36,37,38,39,40,41].

**Figure 1:**
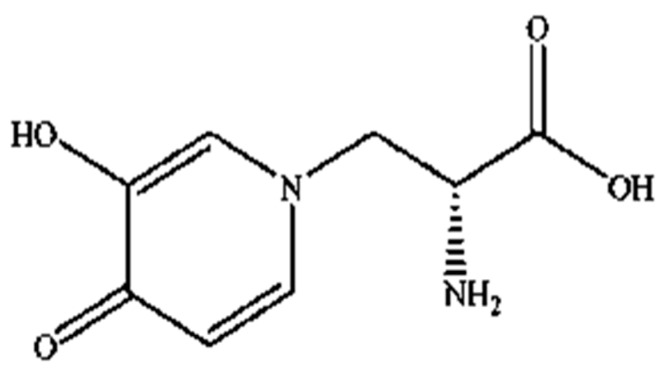
Structure of mimosine.

Mimosine (β-(N-(3-hydroxy-4-oxypyridyl))-α-aminopropionic acid) is a non-protein amino acid present in all members of the *Mimosoideae* family, including LL. Structurally, it contains an alanine side chain bound to the nitrogen atom of a hyroxypyridone ring (Figure 1) with a chemical formula of C_8_H_10_N_2_O_4_ [42,43]. Mimosine possesses several biological properties, including anti-inflammatory [44], anti-viral [45] and anti-cancer properties [46]. It is known as an anti-mitotic agent in many cancer cells, such as pancreatic, prostate, breast, cervical, osteosarcoma, and melanoma cells [36,37,38,39,40,47], where it blocks the G1 phase of the cell cycle and prevents DNA synthesis by inhibiting the formation of the replication fork via deoxyribonucleotide metabolism alteration [40,47,48,49,50]. However, its mechanism of action against parasitic nematodes is unknown, and studies investigating this aspect are still limited.

Due to this, we hypothesized that mimosine could be a potent anthelmintic agent, owing to its toxic nature. Hence, this study aimed to screen and quantify the mimosine content in LL extract and investigate the anthelmintic effects of both LL extract and mimosine on the head thrashing, egg-laying, and pharyngeal pumping activities using the animal model *Caenorhabditis elegans (C. elegans).*

## 2. Results

### 2.1. High-Performance Liquid Chromatography (HPLC) Analysis and Spectrophotometry Revealed the Presence of Mimosine in LL Extract

**Figure 2:**
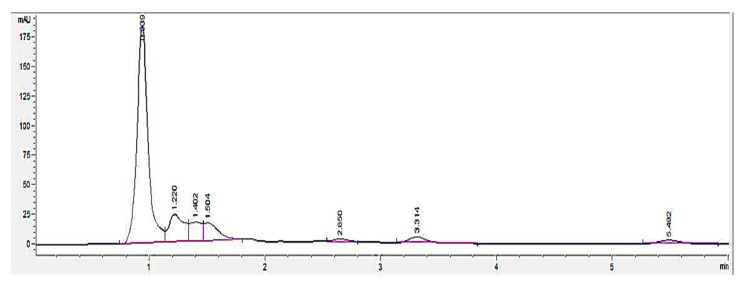
HPLC chromatogram profile for *Leucaena leucocephala* (LL) extract. In total, 7 peaks were revealed at different RTs between 0 to 6 min, with the highest peak at 0.939 min acquiring 184.66 mAU. Key: *y*-axis: mAU, milli-absorbance unit; *x*-axis: min, minutes.

**Figure 3:**
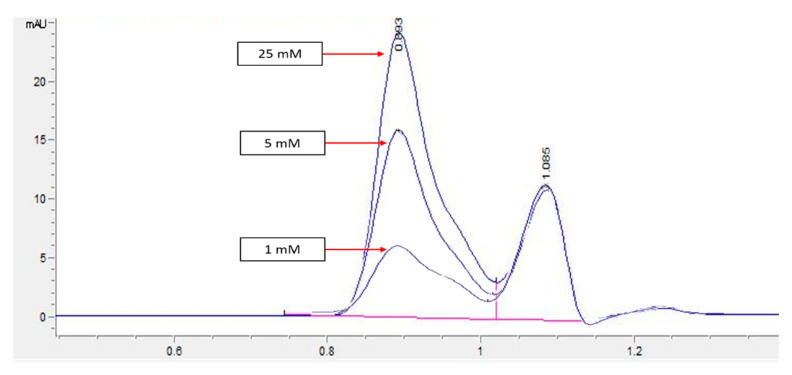
HPLC chromatogram profile for mimosine. Peak heights increased as the concentration of mimosine increased specifically at RT 0.9 min from 7.45 for 1 mM to 24.37 mAU for 25 mM. Key: *y*-axis: mAU, milli-absorbance unit; *x*-axis: min, minutes.

**Figure 4:**
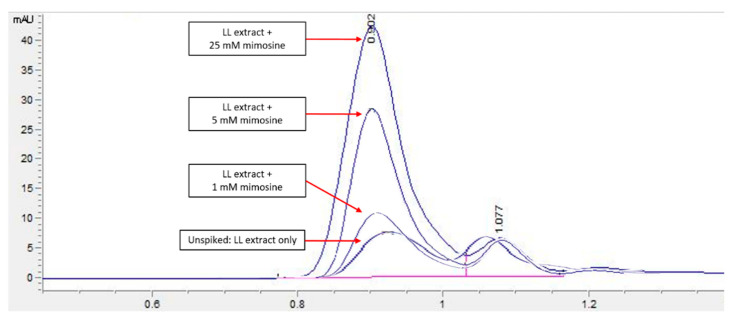
HPLC chromatogram profile for LL extract spiked with mimosine. An increase in the peak heights was apparent for all spiked samples compared to the unspiked LL extract, particularly at an RT of 0.9 min. Key: *y*-axis: mAU, milli-absorbance unit; *x*-axis: min, minutes.

HPLC screening of LL extract alone revealed peaks at retention times (RT) 0.939, 1.220, 1.402, 1.504, 2.650, 3.314, and 5.492 min (mins) (Figure 2). The highest peak was observed at a RT of 0.939 min, with a milli-absorbance unit (mAU) and percentage area of 184.66 mAU and 58.41%, respectively. On the other hand, the HPLC results for the standard reference compound, mimosine, revealed a prominent peak at about 0.9 min for all of the mimosine concentrations. The heights of the peaks were observed to increase at this retention time from 7.45 mAU for 0.2 mg/mL to 24.37 mAU for 5 mg/mL mimosine. Figure 3 shows the chromatogram for mimosine alone, and the HPLC report is tabulated in Table 1. Spiking 200 µg/mL LL extract with the 3 concentrations of mimosine revealed a similar increasing trend in peak heights at the same average RT of 0.9 min compared to the unspiked LL extract. The increment in peak heights was constant, at about 17 mAU between the subsequent spiked samples. The HPLC spiking chromatogram is depicted in Figure 4 and summarized in Table 2.

The mimosine content in the LL extract was determined via spectrophotometry using a standard calibration curve generated from different concentrations of mimosine that ranged between 0.078 to 10 mg/mL using the linear regression equation y = 0.2751x − 0.003987. The spectrophotometric quantification of mimosine revealed approximately 0.119 mg/mL of mimosine in 1 mg/mL of LL extract.

### 2.2. LL Extract and Mimosine Caused Increase in Worm Mortality Dose-Dependently

**Figure 5:**
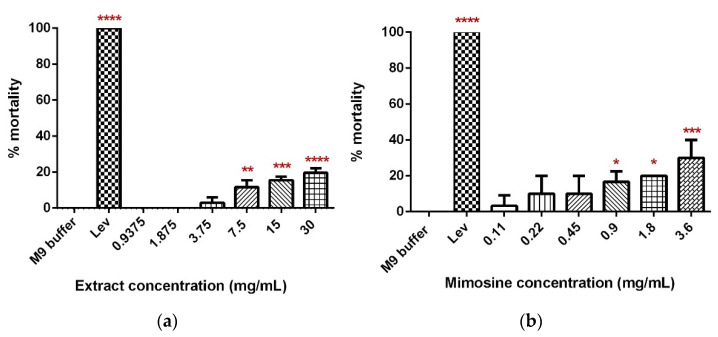
Toxicity of LL extract and mimosine. (**a**) Percentage mortality of *C. elegans* in LL extract. (**b**) Percentage mortality of *C. elegans* in mimosine. Data are presented as means ± SD and are compared to M9 buffer as the negative control. Statistical significance was calculated using a one-way ANOVA with Dunnett’s multiple comparison test and is denoted by asterisks. * *p* ≤ 0.05; ** *p* ≤ 0.01; *** *p* ≤ 0.001; **** *p* ≤ 0.0001. *n* = 30 worms.

A dose-dependent increase in the percentage mortality of *C. elegans* was observed 24 h post-exposure to concentrations of LL extract and mimosine (Figure 5). For LL extract, the percentage of worm death increased from 0% in 0.9375 and 1.875 mg/mL to 19.59 ± 3.73% in 30 mg/mL. Worm mortality was statistically significant for 7.5, 15, and 30 mg/mL of LL extract when compared with the M9 buffer, which did not cause any worm death (Figure 5a). As for mimosine, a significant difference was observed in 0.9, 1.8, and 3.6 mg/mL, and the percentage of mortality increased from 3.33 ± 0.471% in 0.11 mg/mL to 30 ± 0.816% in 3.6 mg/mL (Figure 5b). The LC50 for LL extract and mimosine were estimated from linear regression equations and were calculated to be 73 mg/mL and 6.39 mg/mL (Table 3). The positive control, Lev caused 100% mortality and was significantly different from the M9 buffer.

### 2.3. LL Extract and Mimosine Significantly Decreased Head Thrashing Activity in C. elegans

**Figure 6:**
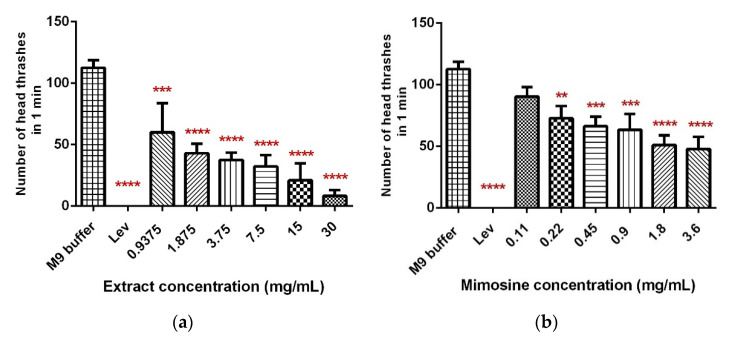
Effect of LL extract and mimosine on head thrashing activity in *C. elegans*. (**a**) The number of head thrashes per min in LL extract. (**b**) The number of head thrashes per min in mimosine. Data are presented as means ± SD and are compared to M9 buffer as the negative control. Statistical significance was calculated using a one-way ANOVA with Dunnett’s multiple comparison test and is denoted by asterisks. ** *p* ≤ 0.01; *** *p* ≤ 0.001; **** *p* ≤ 0.0001. *n* = 15 worms.

The head thrashing activity was significantly reduced in a dose-dependent manner when *C. elegans* were exposed to LL extract and mimosine (Figure 6). Exposure to increasing concentrations of LL extract up to 30 mg/mL caused a significant decrease in the head thrashing movement, from 60 ± 19.37 to 8.13 ± 3.95 thrashes per min (Table 4; Figure 6a). A similar reduction was observed when *C. elegans* were treated with mimosine, whereby the number of head thrashes decreased from 90.4 ± 7.71 at 0.11 mg/mL to 47.27 ± 6.84 thrashes per min at 3.6 mg/mL mimosine (Table 4; Figure 6b). The mean number of head thrashes for most of the concentrations for both treatments was statistically different from the control M9 buffer, which caused a mean thrashing activity of 112.53 ± 5.02 thrashes per min. The positive control, levamisole (Lev) caused complete immobility of the worms.

### 2.4. Significant Inhibition of Egg-Laying Activity in C. elegans Exposed to LL Extract and Mimosine

**Figure 7:**
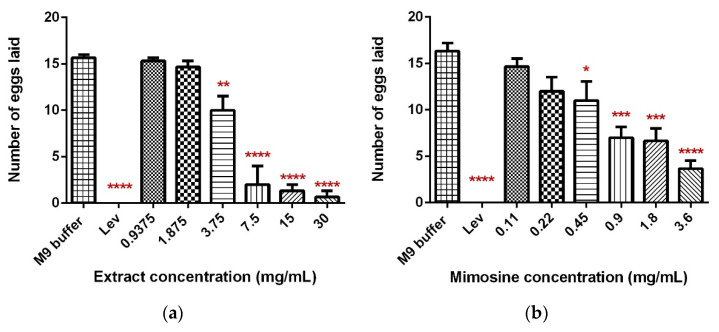
Effect of LL extract and mimosine on the egg-laying activity in *C. elegans*. (**a**) The number of eggs laid in LL extract. (**b**) The number of eggs laid per min in mimosine. Data are presented as means ± SD and are compared to M9 buffer as the negative control. Statistical significance was calculated using a one-way ANOVA with Dunnett’s multiple comparison test and is denoted by asterisks. * *p* ≤ 0.05; ** *p* ≤ 0.01; *** *p* ≤ 0.001; **** *p* ≤ 0.0001. *n* = 15 worms.

The exposure of *C. elegans* to LL extract and mimosine significantly decreased the mean number of eggs laid. The mean number of eggs laid in LL extract reduced substantially to 0.67 ± 0.94 as the concentration increased to 30 mg/mL compared to the M9 buffer control, which had 16.33 ± 1.25 eggs. A significant decrease was also observed when the worms were exposed to mimosine, whereby the highest concentration of 3.6 mg/mL reduced the number of eggs laid to only 3.67 ± 1.25. These results are tabulated in Table 5. Figure 7a,b show the mean number of eggs laid in LL extract and mimosine, respectively.

### 2.5. Mean Pump Amplitude of Pharyngeal Pumping Activity Was Significantly Affected by LL Extract and Mimosine

**Figure 8:**
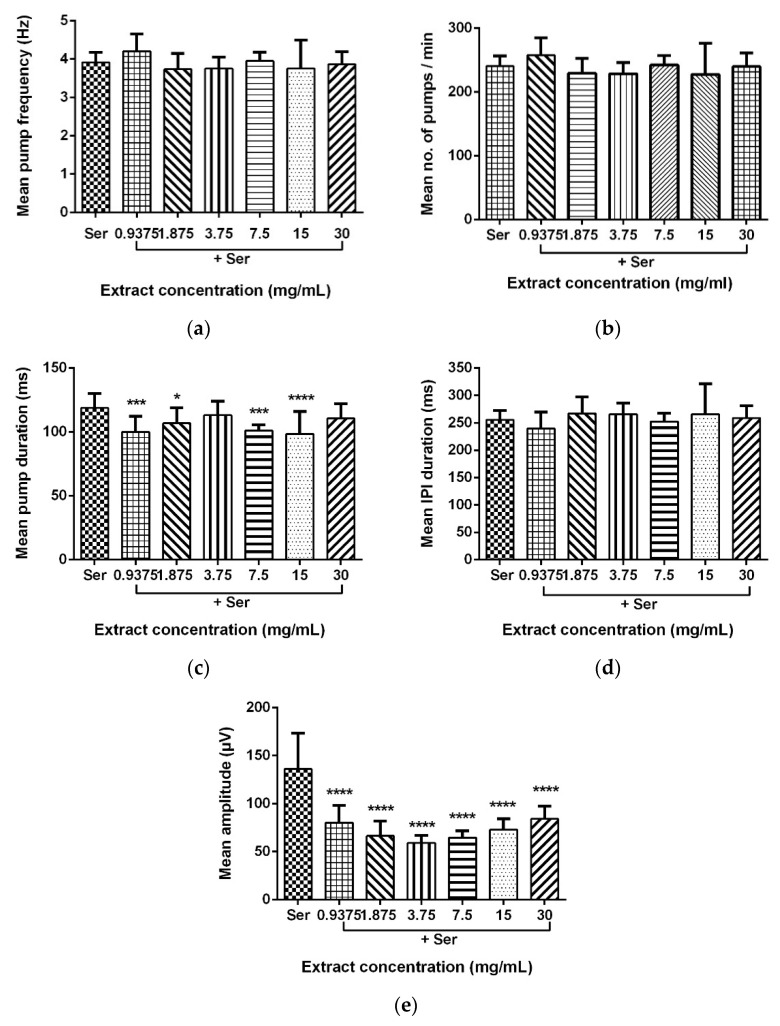
Effect of LL extract on pharyngeal pumping activity. (**a**) Mean pump frequency. (**b**) Mean number of pumps. (**c**) Mean pump duration. (**d**) Mean inter-pump interval (IPI) duration. (**e**) Mean pump amplitude. Data are presented as means ± SD and are compared to Ser as the standard pumping stimulus. Statistical significance was calculated using a two-way ANOVA with Dunnett’s multiple comparison test and is denoted by asterisks. * *p* ≤ 0.05; *** *p* ≤ 0.001; **** *p* ≤ 0.0001. *n* = 15 worms.

**Figure 9:**
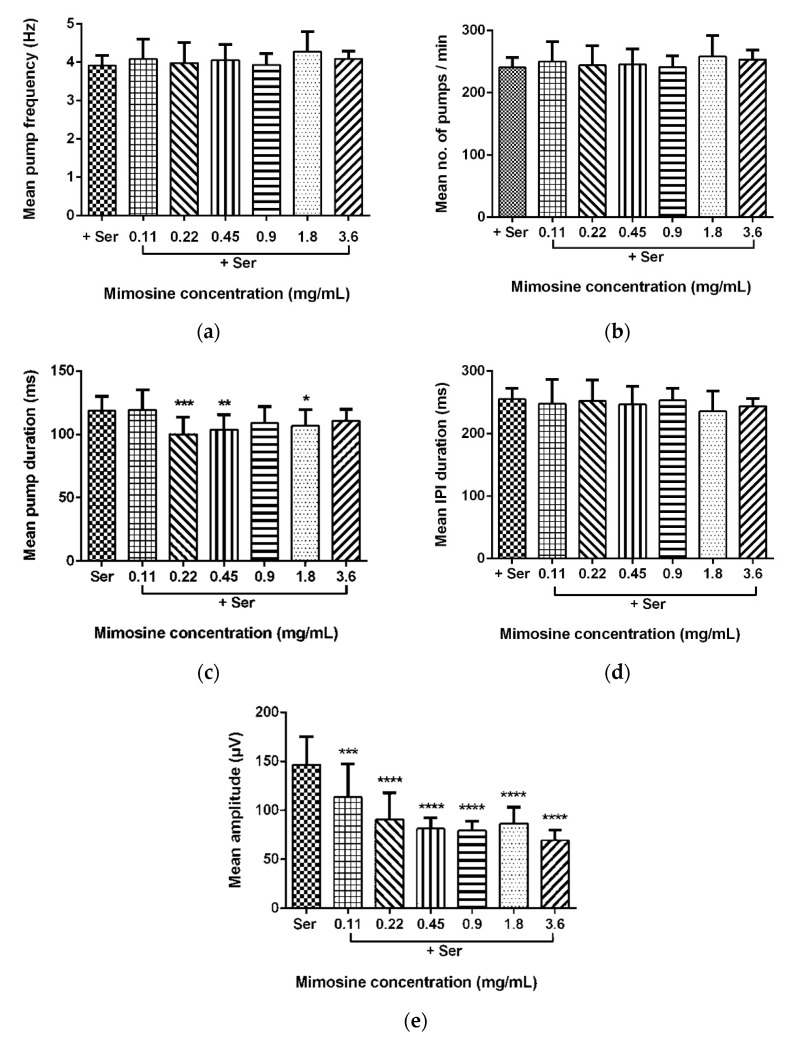
Effect of mimosine on pharyngeal pumping activity. (**a**) Mean pump frequency. (**b**) Mean number of pumps. (**c**) Mean pump duration. (**d**) Mean inter-pump interval (IPI) duration. (**e**) Mean pump amplitude. Data are presented as means ± SD and are compared to Ser as the standard pumping stimulus. Statistical significance was calculated using a two-way ANOVA with Dunnett’s multiple comparison test and is denoted by asterisks. * *p* ≤ 0.05; ** *p* ≤ 0.01; *** *p* ≤ 0.001; **** *p* ≤ 0.0001. *n* = 15 worms.

Data for the mean pump frequency, mean number of pumps, mean pump duration, mean IPI duration, and mean pump amplitude were extracted from the analysis of pharyngeal pumping activity using the NemaMetrix Screenchip^TM^ System. Exposure to different concentrations of LL extract and mimosine significantly decreased the mean pump amplitude. The mean pump amplitude in LL extract was markedly reduced, particularly at concentrations of 3.75 mg/mL to 59.15 ± 7.70 µV compared to those exposed to Ser, which peaked to an average of 146.87 ± 31.53 µV. As for mimosine, the lowest pump amplitude was observed at the highest concentration of 3.6 mg/mL at 69.39 ± 10.14 µV. The other pumping activities were not dramatically affected by both treatments. The results for the pharyngeal pumping activity in LL extract and mimosine are presented in Figure 8 and Figure 9, respectively, and in Table 6.

## 3. Discussion

Several studies have shown the anthelmintic potential of the LL plant [33,41,42,43,44]. The presence of diverse types of bioactive compounds is believed to have contributed to the pharmacological activities in the plant. Several phytochemical candidates are stated in different studies to be responsible for exerting the anthelmintic effect, including flavonoids, tannins, and quercetin [45,46,47,48]. In this study, the phytochemical content of LL extract was screened, and a compound named mimosine was verified to be a potential lead candidate in the extract.

An HPLC analysis of LL extract showed a prominent peak at RT 0.939 min, acquiring the highest height and largest area by percentage. This peak was later identified to represent mimosine based on the HPLC analysis of different concentrations of mimosine that showed an increased peak height at this particular RT as the concentration increased (Figure 3), which was further confirmed by the spiking of LL extract with mimosine that followed the same increasing trend in peak heights at an average of 0.9 min (Figure 4). The second peak in the chromatogram of mimosine alone (Figure 3) might represent a mimosine degradation product or the solvent in which mimosine was dissolved in. The results validate the presence of mimosine in the extract, representing a relatively large proportion compared to the other constituents and opening the possibility of mimosine as the active compound exhibiting pharmacological activities in the LL plant. HPLC analyses of mimosine have been conducted by several studies that showed the mimosine peak to appear at an RT of about 2 to 3 min [35,43,51]. Although these studies do not agree with our finding, this noticeable difference in RTs was mainly due to the use of different mobile phases in these studies, whereby either orthophosphoric acid or potassium dihydrogen phosphate with phosphoric acid and acetonitrile were employed. In this study, acetonitrile and acetic acid were used as the mobile phases. RT values can vary depending on the type of column and the HPLC conditions [35].

Mimosine content in the LL plant is the highest amongst the *Mimosoideae* family. The mimosine content may vary depending on several factors, including plant species, parts of plants, environmental conditions, extraction methods, and type of extracting solvents [34,52,53]. Young leaves and mature seeds contain the highest amount of mimosine (2–10%) compared to the other parts of the plant, such as immature seeds and xylem (0.11–3%) [34,35,43,52]. The quantification of mimosine in the extract in this study by spectrophotometry revealed that the LL extract contained 0.119 mg/mL of mimosine, which is also equal to 0.119 mg/100 g. This finding is more or less concomitant with Sri Wardatun et al. (2020) [53], where the use of 70% ethanol as the solvent extracted 0.37 mg/100 g of mimosine, suggesting that 70% ethanol can be a suitable solvent for the extraction of mimosine from the LL plant. In addition, this is further supported by Benjakul et al. (2014) [52], who demonstrated the highest content of mimosine in the extract that used 60% ethanol compared to using distilled water and 100% and 80% ethanol. The mimosine content may vary depending on several factors, including the species of plant, parts of plants, environmental conditions, extraction methods, and type of extracting solvents [34,52,54].

Many anthelmintic research studies focused on eliminating mimosine from their test candidate or reducing the toxicity of mimosine [34,54]. Only one study has revealed the anthelmintic potential of mimosine whereby the dose-dependent mortality was reported in *C. elegans,* but no further investigation nor insights on the mechanism of action were discussed [42]. Thus, our study could be the first to discover the potential of mimosine as an anthelmintic agent and possibly discover a new mechanism of action. It was already established that mimosine is toxic; similar to other medications or any consumption, it has a dose limit beyond which its action switches from therapeutic to lethal. Therefore, a therapeutic dose for mimosine should be determined in the future so that it causes toxicity to parasitic nematodes but does not impose health complications on the host. However, this would require a broader scope of translational research into an in vivo study using higher organisms and, finally, clinical trial setting.

First, we determined the LC50 for LL extract and mimosine by performing a toxicity assay, exposing increasing concentrations of both samples to *C. elegans* for 24 h. This study demonstrated that LL extract and mimosine significantly increased the percentage of worm mortality in a dose-dependent manner, which generated LC50s of 73 mg/mL and 6.39 mg/mL for LL extract and mimosine, respectively. This shows that our samples had anthelmintic activity. Thus, we then sought to determine how mimosine, as a single compound or in LL extract, affected several behavioral activities in *C. elegans* through performing measurable phenotypic analyses, namely, head thrashing, egg-laying, and pharyngeal pumping assays.

*C. elegans* is highly preferred as an animal model system nowadays, as it is easy to maintain in the lab with minimal nutritional and growth requirements, and it produces a large number of off-springs within a few days, allowing high-throughput assay studies. It has also been fully characterized, with a completely sequenced genome. In addition, *C. elegans* has been a valuable tool in successfully screening and identifying lead compounds for many pharmacological studies, including anthelmintic compounds [55,56,57,58]. Phenotypic read-outs using *C. elegans*, including growth, motility, reproduction, resistance, and survival, were popularly relied on in most in vitro assays to develop anthelmintic drugs and are still today [59,60,61,62]. Screening and identifying drug compounds have become relatively achievable, as these phenotypes are easily measurable via manual or automated assays.

Locomotion in *C. elegans* involves two distinct gaits: swimming or thrashing in liquid and crawling on solid media, which are generally coordinated by the neuromuscular system that produces a rhythmic neuromuscular output [63,64,65]. Forward and reverse motions in *C. elegans* are brought about by rhythmic sinusoidal waves generated from the neuromuscular activity whereby the worm would propel forward when the waves move from tail to head and vice versa [66,67]. The thrashing assay is one of the methods to measure the motility of *C. elegans* that, in turn, serves as an important determining aspect of locomotion. Furthermore, the analysis of thrashing activity can provide valuable insights into identifying the effects of drugs, chemicals, or mutations that affect neuromuscular behavior [68]. In this study, we discovered that LL extract and mimosine negatively affected the head thrashing movement in *C. elegans.* Significant dose-dependent decreases in the mean number of head thrashes were observed in worms exposed to increasing concentrations of LL extract (0.9875, 1.875, 3.75, 7.5, 15, and 30 mg/mL) and mimosine (0.11, 0.22, 0.45, 0.9, 1.8, and 3.6 mg/mL). Our findings imply that LL extract or mimosine possess an anthelmintic activity that significantly alters head thrashing movements.

Next, changes in the egg-laying behavior in *C. elegans* is also one of the reliable, quantifiable neuromuscular outputs along with locomotion, feeding, and defecation [69]. The egg-laying machinery is composed of 16 vulval muscles and two types of neurons: hermaphrodite-specific neurons (HSNs) and ventral type C neurons (VCs) [69,70,71,72,73]. The neurons synapse to the vulval muscles, causing muscle contraction and the opening of the vulva that eventually leads to the expulsion of eggs into the environment. The egg-laying activity in this study was also found to be significantly reduced after exposure to LL extract and mimosine, as demonstrated by the decrease in the mean number of eggs laid. Similar research findings concordantly showed the inhibition of egg-laying and -hatching post-treatment with LL extracts in different nematode species [30,32,74,75,76,77,78,79,80]. For instance, a recent study by Romero et al. (2020) [76] reported that the ethanolic extract of LL leaves significantly inhibited egg-hatching activity up to 54% in *Haemonchus contortus.* These studies strongly support the ability of LL plant extracts to inhibit the laying and hatching of eggs in nematodes.

Pharyngeal pumping is one of the motions in *C. elegans* feeding process. It is the near-simultaneous contraction and relaxation of the muscles in the pharynx, namely, the corpus, anterior isthmus, and terminal bulb [81,82]. The pumping rate of the pharynx depends on the surrounding environment, where in the abundance of food or the presence of an exogenous or endogenous pumping stimulus, such as serotonin, the rate would be at its optimum of 200–300 pumps per min [71,81,83]. In contrast, the pump would become slower to about 50–100 pumps per min when food is scarce. Proper timing and coordination of pharyngeal pumping are vital to ensure efficient feeding and, hence, the overall state of health of *C. elegans.* The pharyngeal pumping process is coordinated by neuromuscular activity that leads to the generation of an action potential. This action potential is derived from the activity of different ion channels involved in pharyngeal pumping, which correspond to patterns observed in the EPG diagram. In brief, activating a nicotinic acetylcholine (ACh) receptor called EAT-2 via ACh binding initiates the pumping action. After reaching a certain membrane potential threshold, a second channel, CCA-1 is activated. CCA-1 is a T-type calcium channel that causes an influx of calcium ions upon activation, which accelerates the action potential upstroke in the pharyngeal muscles. An L-type calcium channel called EGL-19 is activated by an increase in membrane potential from the CCA-1 channel. Upon opening the channel, it allows more influx of calcium ions and further depolarizes the muscle, leading to the contraction motion of the pharyngeal muscles [84,85].

We have discovered that LL extract and mimosine reduced the mean pump amplitude of pharyngeal pumping, evidenced from the analysis of EPG recordings via NemAnalysis software. It was also found that the other pumping parameters, the number of pumps, frequency, duration, and IPI duration, did not change compared to the control, Ser. This finding has provided valuable insight into the possible mechanism of action exhibited by LL extract and mimosine.

Pump amplitude in the pharyngeal pumping activity represents the strength of contraction of the pharyngeal muscles. A higher amplitude means a stronger contraction. Since the contraction of pharyngeal muscles is potentiated by the EGL-19 channel activity described above, we hypothesized that LL extract and mimosine might act as a calcium channel blocker or inhibitor, mainly targeting the EGL-19 channel.

EGL-19 is a voltage-gated (VGCC), L-type calcium channel that primarily functions as an entry route for the influx of Ca^2+^ ions into muscle cells, which would lead to muscle depolarization and, eventually, muscle contractions [86]. Most neuromuscular activity in *C. elegans* is reported to be sensitive to the activity of a sole gene, which is *egl-19,* that encodes the pore-forming α1 subunit of the L-type VGCC [87,88]. This gene and, hence, the EGL-19 channel is functionally the major VGCC in muscles of *C. elegans* expressed in different parts of the worm, including the alimentary muscles, pharyngeal muscles, body wall muscles, neurons, and preanal ganglion, which contribute to the function of EGL-19 in the development, feeding, egg-laying, mating, and locomotion [84,87,88,89,90,91]. In addition, the reduction-of-function allele of *egl-19* has been shown to affect the excitation of the vulval, body wall, and pharyngeal muscles [92,93,94]. This further supports our hypothesis of the inhibitory action of LL extract and mimosine on the EGL-19 channel, which would explain the suppression of the head thrashing, egg-laying, and pharyngeal pumping activities observed in this study. Hence, it is imperative to perform further research in the future to validate this hypothesis.

## 4. Materials and Methods

### 4.1. Chemicals and Reagents

All chemicals used for the extraction, high-performance liquid chromatography (HPLC), and quantification of mimosine content were obtained from Merck, Millipore, Germany. The activated carbon for determination of mimosine content, L-mimosine (mimosine), and serotonin (Ser) used in the pharyngeal pumping assay were purchased from Sigma-Aldrich, St. Louis, MO, USA. For worm synchronization, sodium hypochlorite and sodium hydroxide (NaOH) were obtained from The Clorox Company, Oakland, CA, USA and Merck, Millipore, Germany.

### 4.2. Plant Collection and Authentication

Mature LL plant was collected in the compounds of The Core Building and PAPRSB Institute of Health Sciences (IHS), Universiti Brunei Darussalam (UBD). The plant was sent for species identification and deposited in UBD Herbarium (UBDH) with the identification number 17H0176. Dr Johan Willem Federik Slik, Associate Professor, Faculty of Science, UBD and curator of UBDH verified the authenticity of the plant.

### 4.3. Preparation of LL Extract and Mimosine

LL extract was prepared as described previously with slight modifications [95]. In brief, the mature seeds of LL were first washed to remove impurities and oven-dried at 40 °C for about 3–4 days. The seeds were ground to a fine powder, macerated in 70% ethanol (1:10 *v*/*v* solute to solvent) with constant stirring for two hours, and left for a 24 h incubation at 37 °C in a shaking incubator. The extract was then filtered using Whatman No. 1 filter paper (125 mm). The filtrate was dried in an oven at 40 °C to yield a sticky dark brown LL crude extract and stored at −4 °C until further use. A range of concentrations (0.9375, 1.875, 3.75, 7.5, 15, and 30 mg/mL) was prepared for experimental assays. For mimosine, a range of concentrations (0.11, 0.22, 0.45, 0.9, 1.8, and 3.6 mg/mL) was prepared in distilled water.

### 4.4. HPLC Analysis of LL Extract Content and Validation of the Presence of Mimosine

HPLC analysis was performed following the procedure as previously described [96] using an Agilent 1200 Series HPLC System (Agilent LabX, Santa Clara, CA, USA) to detect the active compounds in the extract and validate the presence of mimosine. The mobile phases used consisted of 5% acetic acid (A) and 95% acetonitrile (B) run through a reverse phase C18 column (Sigma-Aldrich, USA) (internal diameter: 4.6 mm; height: 250 mm; particle size: 5 mm) with the following elution program: 0–10 min, 70% A isocratic, 10–20 min gradient to 40% A, and 20–30 min 40% A isocratic. The program was set at a 1.0 mL/min flow rate at 30 °C with an injection volume of 20 µL. The analysis was run for 60 min, and the data acquisition was performed at a 367 nm wavelength. The LL extract and mimosine were filtered through a 0.22 µm syringe-driven filter (Merck Millipore, Germany) to avoid the blockage of the column. LL extract alone (1 mg/mL) was first run to analyze the different compound fractions present in the extract. In order to determine the peak that represents mimosine, the standard compound mimosine was used at concentrations of 0.2, 1, and 5 mg/mL, followed by running LL extract (200 µg/mL) spiked with the different concentrations of mimosine.

### 4.5. Quantification of Mimosine Content by Spectrophotometry

The determination of mimosine content in the LL extract was conducted as described previously [97]. The extract (50 mg/mL) was first dissolved in distilled H_2_O, and 0.6 mg of activated carbon (Sigma-Aldrich, USA) was added. The mixture was boiled and filtered using a 0.45 µm syringe-driven filter (Thermo Fisher Scientific, Dongguan, China). About 500 µL of 0.1 N HCl and 200 µL of the mixture was added into a microfuge tube, followed by the addition of 400 µL of 0.1 N HCl and 200 µL of 0.5% FeCl_3_ in 0.1 N HCl. The mixture’s 100 µL aliquots were transferred into respective wells in a 96-well plate. The absorbance was then measured at 534 nm using an Epoch 2 Microplate Reader (Biotek Company, Minneapolis, MN, USA). Different concentrations of mimosine between 0.078 and 10 mg/mL in serial dilutions were used as the calibration standard. The quantified mimosine content was expressed in mg/100 g.

### 4.6. Maintenance of C. elegans and Synchronization

Wild-type N2 strain *C. elegans* (Bristol) was acquired from the Caenorhabditis Genetics Centre (CGC), University of Minnesota, USA. The worms were grown on nematode growth medium (NGM) agar plates seeded with *Escherichia coli* (*E. coli*) OP50 bacteria at 22 °C. Worm synchronization was performed as previously explained with few modifications [98]. Agar plates with adequate populations of eggs or hermaphrodite worms were washed with M9 buffer and transferred into a 50 mL falcon tube. The tube was centrifuged at 1500 rpm for 1 min, and the supernatant was discarded. In order to kill the adult worms and isolate the eggs only, an alkaline bleaching solution was added (a mixture of sodium hypochlorite and 2 M NaOH at a ratio of 1:1 (*v*/*v*)). The tube was shaken vigorously for 3 min before adding 10 mL of M9 buffer to stop the bleaching effect. After centrifugation again at 1500 rpm for 1 min, the supernatant was discarded, followed by the addition of 10 mL of M9 buffer. This procedure was repeated until the solution was clear of bacteria or dead worm debris. About 1 mL of M9 buffer was added for the final centrifugation, and the supernatant was discarded, leaving only approximately 100 µL of egg pellets, which were transferred onto an unseeded NGM plate. The eggs were hatched to L1 larvae, then transferred into a seeded agar plate, and grown to the desired life stage.

### 4.7. Toxicity Assay

First, 50 µL of each of the following were added into allocated wells in a 96-well plate: LL extract in increasing concentrations (0.9375, 1.875, 3.75, 7.5, 15, and 30 mg/mL) prepared in M9 buffer or mimosine (0.11, 0.22, 0.45, 0.9, 1.8, and 3.6 mg/mL), M9 buffer (negative control), and 100 µM levamisole (Lev) (positive control) (Sigma-Aldrich, USA). Next, 10 synchronized L4-staged worms were then added into each treatment and incubated at 22 °C for 24 h. The number of worm deaths was then observed using a dissecting microscope (Motic SM2-140 Series, Carlsbad, CA, USA) and recorded. Dead worms were characterized by having straight bodies and not responding upon being touched or disturbed using a metal wire. The assay was conducted in triplicate. The LC50 values were determined from linear regression equations using GraphPad Prism (Version 6.01, San Diego, CA, USA).

### 4.8. Head Thrashing Assay

This assay was performed as follows, with slight modifications [99]. About 50 µL of each extract and mimosine concentrations were added into allocated wells in a 96-well plate. M9 buffer and 100 µM Lev acted as negative and positive controls. Five synchronized L4 worms were placed in each well and incubated for 2 h at 22 °C. The number of head thrashes was counted for 30 s and doubled to obtain the thrashing activity of 1 min. The assay was performed in triplicate.

### 4.9. Egg-Laying Assay

About 50 µL of each of the extract and mimosine concentrations were added into allocated wells in a 96-well plate, with M9 buffer and 100 µM levamisole acting as the respective negative and positive controls. Next, 5 synchronized gravid hermaphrodites were then added into each treatment and incubated at 22 °C for 4–6 h to allow the laying of eggs. The number of eggs laid and hatched was then counted. The assay was performed in triplicate.

### 4.10. Pharyngeal Pumping Assay

Synchronized young adult worms were washed with M9 buffer and pelleted by centrifugation at 1500 rpm for 1 min. The supernatant was discarded, and the worm pellet was resuspended with 1 mL of M9 buffer and centrifuged again. This step was repeated three times. The final supernatant was discarded, leaving only about 100 µL of the worm suspension, which was transferred to an unseeded NGM plate, allowing the worms to starve for about two hours before running the experiment. After being subjected to starvation, the worms were resuspended in M9 buffer into a microfuge tube. The worms were then exposed to different concentrations of LL extract or mimosine with initial pumping induction with 10 mM Ser. The worms were allowed to acclimate for 10 min after incorporating the drugs before being pumped into a NemaMetrix Screenchip SC40 (InVivo Biosystems, Eugene, OR, USA) using a syringe. The Screenchip was first flushed with M9 buffer three times to remove bubbles and debris before pumping in the treated worms. Electropharyngeogram (EPG) recordings were taken using the NemaMetrix Screenchip^TM^ System (InVivo Biosystems, Eugene, OR, USA) for a period of 1 min through the NemAcquire Software (InVivo Biosystems, Eugene, OR, USA). Only worms with observable pumping activity were taken into record. The EPGs were analyzed using the NemAnalysis Software. The mean number of pumps, mean pump frequency, mean pump duration, mean inter-pump-interval (IPI) duration, and mean pump amplitude were plotted. A total of 15 worms were taken into the finalized data.

### 4.11. Statistical Analysis

Statistical analyses were performed using GraphPad Prism (Version 6.01) software (GraphPad Software, Inc., San Diego, CA, USA). The data between the experimental treatments and the control in the head thrashing and egg-laying assays were analyzed using a one-way analysis of variance (ANOVA) with Dunnett’s multiple comparison test, while a two-way ANOVA with Dunnett’s multiple comparison test was used for the pharyngeal pumping data. The data were expressed as means ± standard deviations (SD). Statistical significance was recorded as * *p* ≤ 0.05; ** *p* ≤ 0.01; *** *p* ≤ 0.001; **** *p* ≤ 0.0001. 

## 5. Conclusions

The ethanolic extract of LL mature seeds has been shown to exhibit anthelmintic effects in *C. elegans*, and an active compound named mimosine has been identified as the potential lead candidate in this extract responsible for the activity. LL extract and mimosine significantly decreased the head thrashing and egg-laying activities and the mean pump amplitude, representing the strength of pharyngeal pumping in *C*. *elegans*. The findings in this study have pointed to the possible inhibition of an L-type calcium channel called EGL-19 by LL extract and mimosine, which may act as calcium channel antagonists. Further investigation is necessary to confirm this hypothesis.

## Figures and Tables

**Table 1:** HPLC report for mimosine.

Mimosine (mg/mL)	Retention Time (RT) (min)	Height (mAU)	Area (mAU X s)	Area (%)
0.2	0.908	7.45316	42.06311	50.3087
	1.086	10.97289	41.54688	49.6913
1	0.9	16.02594	87.79227	67.1053
	1.088	11.14823	43.03543	32.8947
5	0.893	24.3674	132.21669	74.4095
	1.085	11.47292	45.47133	25.5905

**Table 2:** HPLC report for LL extract with and without spiking with mimosine.

Samples		Retention Time (RT) (min)	Height (mAU)	Area (mAU X s)	Area (%)
**Unspiked**		0.908	7.25016	49.88125	52.6444
**Spiked**	LL extract + 0.2 mg/mL mimosine	0.896	9.15798	54.45244	70.4267
	LL extract + 1 mg/mL mimosine	0.886	26.47382	140.31894	84.1147
	LL extract + 5 mg/mL mimosine	0.902	42.3965	238.33374	88.5571

**Table 3:** Estimated LC50 values of LL extract and mimosine.

Treatments	Regression Equation	LC50 Value
LL extract	Y = 0.6939X + 1.461	73 mg/mL
Mimosine	Y = 6.713X + 7.097	6.39 mg/mL

**Table 4:** Head thrashing activity of LL extract and mimosine.

Treatments	Concentration (mg/mL)	Mean Number of Head Thrashes per min ± Standard Deviation (SD)
M9 buffer (negative control)		112.53 ± 5.02
Levamisole (Lev) (positive control)	100 µM	0 ****
LL extract	0.9875	60 ± 19.37 ***
	1.875	42.93 ± 6.37 ****
	3.75	37.6 ± 4.83 ****
	7.5	32.13 ± 7.70 ****
	15	20.93 ± 11.21 ****
	30	8.13 ± 3.95 ****
Mimosine	0.11	90.40 ± 7.71
	0.22	74.67 ± 6.58 **
	0.45	64.80 ± 9.62 ***
	0.9	63.47 ± 22.03 ***
	1.8	51.27 ± 7.56 ****
	3.6	47.27 ± 6.84 ****

** *p* ≤ 0.01; *** *p* ≤ 0.001; **** *p* ≤ 0.0001.

**Table 5:** The egg-laying activity of LL extract and mimosine.

Treatments	Concentration (mg/mL)	Mean Number of Head Thrashes per min ± Standard Deviation (SD)
M9 buffer (negative control)		16.33 ± 1.25
Lev (positive control)	100 µM	0 ****
LL extract	0.9875	15.33 ± 0.47
	1.875	14.67 ± 0.94
	3.75	10 ± 2.16 **
	7.5	2 ± 2.83 ****
	15	1.33 ± 0.94 ****
	30	0.67 ± 0.94 ****
Mimosine	0.11	14.67 ± 1.25
	0.22	12 ± 2.16
	0.45	11 ± 2.94 *
	0.9	7.33 ± 1.25 ***
	1.8	6 ± 1.63 ***
	3.6	3.67 ± 1.25 ****

* *p* ≤ 0.05; ** *p* ≤ 0.01; *** *p* ≤ 0.001; **** *p* ≤ 0.0001.

**Table 6:** Pharyngeal pumping activity of LL extract and mimosine.

Treatments	Concentrations (mg/mL)	Pharyngeal Pumping Parameters				
		Mean Pump Frequency (Hz)	Mean Number of Pumps/min	Mean Pump Duration (ms)	Mean Inter-Pump Interval (IPI) Duration (ms)	Mean Pump Amplitude (µV)
**Ser alone (control)**	10 mM	240.67 ± 15.16	3.91 ± 0.25	118.87 ± 10.75	255.22 ± 16.47	146.87 ± 31.53
**LL extract**	0.9875	257.73 ± 26.31	4.20 ± 0.45	99.83 ± 11.89 ***	293.73 ± 28.74	80.16 ± 17.59 ****
	1.875	229.27 ± 22.21	3.74 ± 0.40	106.86 ± 11.77 *	226.89 ± 29.65	66.42 ± 15.06 ****
	3.75	228.73 ± 16.48	3.75 ± 0.28	113.16 ± 10.38	265.75 ± 19.44	59.16 ± 7.70 ****
	7.5	242.33 ± 14.08	3.95 ± 0.23	101.09 ± 4.19 ***	252.44 ± 14.66	64.52 ± 6.75 ****
	15	227.40 ± 47.68	3.76 ± 0.72	98.28 ± 17.09 ****	265.98 ± 53.46	72.74 ± 11.23 ****
	30	239.8 ± 20.70	3.86 ± 0.32	110.50 ± 11.04	258.83 ± 21.41	84.04 ± 12.91 ****
**Mimosine**	0.11	245.73 ± 36.65	3.99 ± 0.58	119.43 ± 15.29	255.09 ± 42.93	113.45 ± 33.094 ****
	0.22	244.20 ± 30.45	3.98 ± 0.52	100.21 ± 12.83 ***	252.31 ± 32.75	90.84 ± 26.31 ****
	0.45	245.53 ± 24.20	4.05 ± 0.40	103.74 ± 11.37 **	246.79 ± 27.57	81.40 ± 10.67 ****
	0.9	241.27 ± 17.83	3.93 ± 0.28	108.77 ± 12.73	253.22 ± 18.56	79.41 ± 9.31 ****
	1.8	258.40 ± 32.52	4.28 ± 0.50	106.56 ± 12.55 *	235.98 ± 30.77	90.78 ± 21.26 ****
	3.6	253.27 ± 14.59	4.09 ± 0.20	110.76 ± 8.72	243.89 ± 11.86	69.39 ± 10.14 ****

* *p* ≤ 0.05; ** *p* ≤ 0.01; *** *p* ≤ 0.001; **** *p* ≤ 0.0001.

## Data Availability

Not applicable.
